# Increased wood biomass growth is associated with lower wood density in *Quercus petraea* (Matt.) Liebl. saplings growing under elevated CO_2_

**DOI:** 10.1371/journal.pone.0259054

**Published:** 2021-10-22

**Authors:** Janko Arsić, Marko Stojanović, Lucia Petrovičová, Estelle Noyer, Slobodan Milanović, Jan Světlík, Petr Horáček, Jan Krejza

**Affiliations:** 1 Global Change Research Institute of the Czech Academy of Sciences, Brno, Czech Republic; 2 Department of Forest Ecology, Faculty of Forestry and Wood Technology, Mendel University in Brno, Brno, Czech Republic; 3 Faculty of Forestry, University of Belgrade, Belgrade, Serbia; 4 Department of Forest Protection and Wildlife Management, Faculty of Forestry and Wood Technology, Mendel University in Brno, Brno, Czech Republic; 5 Department of Wood Science and Technology, Faculty of Forestry and Wood Technology, Mendel University in Brno, Brno, Czech Republic; Universidade Federal de Alfenas, BRAZIL

## Abstract

Atmospheric carbon dioxide (CO_2_) has increased substantially since the industrial revolution began, and physiological responses to elevated atmospheric CO_2_ concentrations reportedly alter the biometry and wood structure of trees. Additionally, soil nutrient availability may play an important role in regulating these responses. Therefore, in this study, we grew 288 two-year-old saplings of sessile oak (*Quercus petraea* (Matt.) Liebl.) in lamellar glass domes for three years to evaluate the effects of CO_2_ concentrations and nutrient supply on above- and belowground biomass, wood density, and wood structure. Elevated CO_2_ increased above- and belowground biomass by 44.3% and 46.9%, respectively. However, under elevated CO_2_ treatment, sapling wood density was markedly lower (approximately 1.7%), and notably wider growth rings—and larger, more efficient conduits leading to increased hydraulic conductance—were observed. Moreover, despite the vessels being larger in saplings under elevated CO_2_, the vessels were significantly fewer (*p* = 0.023). No direct effects of nutrient supply were observed on biomass growth, wood density, or wood structure, except for a notable decrease in specific leaf area. These results suggest that, although fewer and larger conduits may render the xylem more vulnerable to embolism formation under drought conditions, the high growth rate in sessile oak saplings under elevated CO_2_ is supported by an efficient vascular system and may increase biomass production in this tree species. Nevertheless, the decreased mechanical strength, indicated by low density and xylem vulnerability to drought, may lead to earlier mortality, offsetting the positive effects of elevated CO_2_ levels in the future.

## 1. Introduction

Global atmospheric CO_2_ concentration has increased by more than 45% since the industrial revolution began, reaching above 410 ppm in 2020 [1]. By and large, this substantial increase has been caused by CO_2_ released from anthropogenic emissions, such as the burning of fossil fuels, deforestation, and other land-use changes. Currently, the average rate of increase in atmospheric CO_2_ concentration is 2 ppm per year, whereby the global atmospheric CO_2_ level is likely to increase to 550–1000 ppm by the end of the century [2], depending on the success of our efforts to reduce CO_2_ emissions [3]. Further, elevated CO_2_ concentrations (hereafter, eCO_2_) are the main cause of global warming and are predicted to cause an increase in air temperature from 2 to 5 °C by the end of the 21^st^ century [3]. Through photosynthesis, forests sequester approximately 26% of anthropogenic carbon emissions each year [4], thus playing an important role in preventing global warming. Additionally, an increase in anthropogenic nitrogen deposition enhances soil nitrogen availability, which may reportedly hinder the CO_2_-sequestering ability of plants [5, 6].

Numerous studies have demonstrated the effects of eCO_2_ levels on plant growth and function over the last few decades [7–12], wherein increased atmospheric CO_2_ levels were shown to improve tree growth through a process known as the “fertilisation effect”. Photosynthesis is directly influenced by variations in CO_2_ concentrations and is one of the most studied physiological processes. Previous studies have shown that at the leaf level, eCO_2_ concentrations increase photosynthesis rates while reducing stomatal conductance, thereby enhancing water use efficiency (WUE) [13–16]. The carbohydrates produced in the process serve as the building blocks for plant biomass production and many other processes in which carbon is metabolised [17]. Therefore, the enhancement of photosynthesis and the resulting carbohydrate surplus promoted by eCO_2_ levels lead to a consistent increase in biomass growth. However, these effects have been mostly observed in young saplings [10, 14, 18], whereas mature trees usually do not show a positive growth response to eCO_2_ levels [19]. In addition, growth stimulation by eCO_2_ may be limited by the progressive scarcity of soil nutrients, particularly nitrogen [20–24]. Indeed, the effects of N deficiency on eCO_2_-induced growth stimulation are evident in plants growing on severely nutrient-deficient substrates [25]. Thus, any generalisation of plant responses to nutrient unavailability under eCO_2_ levels is difficult to make [13].

Although previous studies have investigated the effects of eCO_2_ levels on plant physiological and growth responses, few studies have addressed the changes in wood structure resulting from elevated CO_2_ concentrations [26]. Furthermore, the changes in the physiology and growth of a plant not only alter the internal water balance [27] but also affect the structure and functioning of tracheids and vessel elements in the secondary xylem [28, 29] as well as xylem hydraulic conductance [30]. Nonetheless, the effects of eCO_2_ levels on xylem elements vary among species. For instance, the tracheid lumen area in coniferous species such as *Larix sibirica* Ledeb. [31] and *Pinus sylvestris* L. [29] increased under eCO_2_ levels. In contrast, diffuse-porous tree species were less responsive to eCO_2_ levels and no effect was observed on the vessel area in *Populus tremuloides* Michx. [32], *Betula pendula* Roth. [33], or *Fagus sylvatica* L. [34]. Moreover, the lesser-investigated ring-porous tree species showed inconsistent results under eCO_2_ levels, for example, increased vessel area in *Quercus robur* L. [35] but no changes in *Quercus mongolica* Fisch. Ex Ledeb. [28, 36]. Nevertheless, Watanabe et al. [28] also observed wider vessels and higher hydraulic conductance in *Q*. *mongolica* trees grown in N-rich soil regardless of CO_2_ concentration.

The lumen area of xylem conduits is an important factor that influences not only the water flux capacity of xylem [37] but also wood density. Wood density is a key determinant of plant ecological strategies [38], including both hydraulic and mechanical strategies, as denser wood is more resistant to cavitation and is stiffer and less susceptible to wind damage [39]. Increased plant growth rate is another wood density-related factor associated with lower wood density [40–42] that renders fast-growing trees more susceptible to high winds [43]. Information about the effect of eCO_2_ on wood density is rather scarce in the literature. Conifers are known to exhibit higher wood density under eCO2, owing to their characteristically higher proportion of latewood. In contrast, angiosperms show no changes in wood density under eCO_2_ levels [26]; however, they have been much less studied.

Therefore, with the simultaneous increase in CO_2_ levels and nitrogen deposition in the coming decades, forest ecosystems will face many challenges that will force plants to adapt to warmer temperatures, higher evaporative demand, and increased frequency and severity of drought events [44]. Nonetheless, as difficult as it may seem to predict how forests will respond to these changes, the range of tree species in Central Europe will likely be substantially altered from the presently dominant conifers, to angiosperms, particularly thermophilous oak species [45, 46].

Sessile oak (*Quercus petraea* (Matt.) Liebl.), a broad-leaved, ring-porous tree species, is widespread in the temperate zone and is one of the most ecologically and economically important hardwood tree species in Central Europe [47, 48]. Owing to the morphological (deep rooting, leaf curling, and leaf loss) and physiological (osmotic adjustment and stomatal control to reduce transpiration water loss) mechanisms to overcome drought stress [49, 50], sessile oaks are considered well adapted for future climate scenarios [51, 52].

Therefore, the objective of this study was to investigate the effects of (i) eCO_2_ concentration and (ii) nutrient supply on biomass growth, wood density, and wood structure of the conductive tissue in sessile oak saplings. Our main hypothesis was that the growth of sessile oak saplings will be stimulated by the fertilisation effect of eCO_2_. Previous studies have reported that higher growth rates in sessile oak in Western and Central Europe are associated with lower wood density [41, 42, 53]; accordingly, we tested the hypothesis that eCO_2_ concentration is the cause for the observed morphological difference. Furthermore, it was expected that the higher growth rates would be followed by altered wood anatomy, which in turn would be reflected in a larger more efficient conductive system. Finally, we also hypothesised that the magnitude of the CO_2_ fertilisation effect is regulated by nutrient supply.

## 2. Materials and methods

### 2.1. Study area

The experiment was conducted in two neighbouring glass domes (area: 10 × 10 m^2^; central height: 7 m) in the Bílý Kříž experimental ecological station situated in the Moravian-Silesian Beskydy mountains (49°30’77″ N, 18°32’28″ E; Elevation: 908 m asl). The mean air temperature and the mean precipitation during the period 1998–2014 were 6.8 °C and 1,260 mm, respectively. The soil type is ferric podzol, overlying the Mesozoic Godula sandstone (flysch-type), which is moderately rich and has a high humus content (5%–7%).

Under the scenario that has most closely tracked human historical emission trajectories, the atmospheric CO_2_ concentrations will reach approximately 550 to 700 ppm by the middle-to-late 21st century [2, 3]. Previous studies on the effect of elevated CO_2_ on different tree species were conducted mostly based on these concentrations [16, 54, 55], whereas more pessimistic predictions of 900–1000 ppm, which are expected by the end of 21 century, have been rarely used [13, 18]. In our study, ambient CO_2_ (hereafter aCO_2_) and eCO_2_ treatment concentrations in the open-top glass domes were 400 and 700 ppm, respectively, and CO_2_ was continuously supplied from April to November, from 2017 to 2019. The design and installation of the glass domes was as described previously [56]. The microclimatic conditions inside the two domes were maintained at similar levels, as shown by the non-significant differences in temperature (*p* = 0.953) and relative air humidity (*p* = 0.485). The soil inside the glass domes was natural and not mechanically altered, representing the same soil type as at the study site.

**Fig 1:**
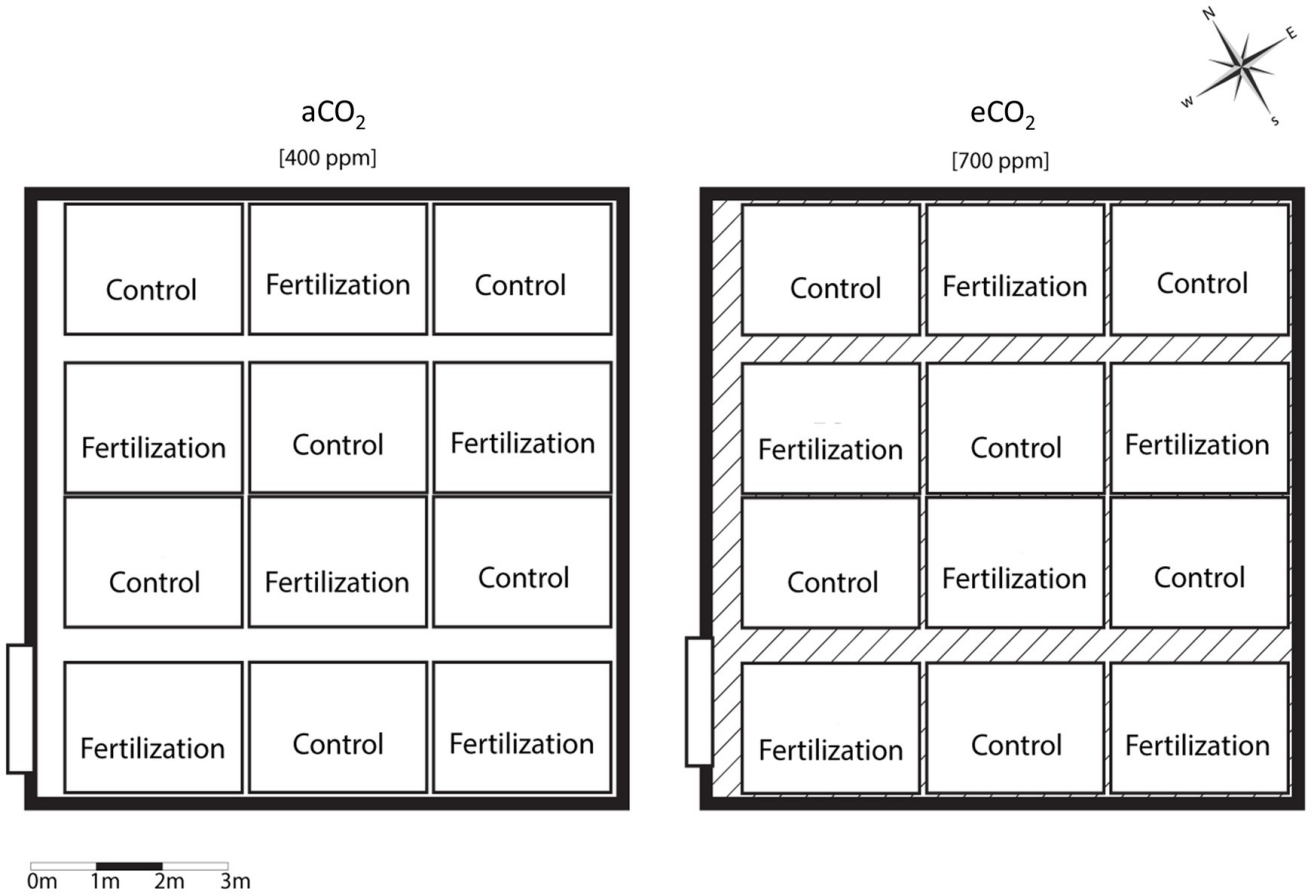
Experimental design scheme. aCO_2_—ambient CO_2_ concentration, eCO_2_—elevated CO_2_ concentration, Control—plots without nutrient supply, Fertilization—plots with nutrient supply.

A total of 144 two-year-old sessile oak saplings were planted in each glass dome in 2017, following a specific fertilisation experimental design (Fig 1). Each glass dome was split into twelve blocks: no fertiliser was added to six blocks (control), while the other six blocks were annually supplied with calcium nitrate (AGRO, Czech Republic) containing 15% nitrogen, at a rate of 5 g m^-2^ year^-1^ in mid-April during the study period (2018–2019).

### 2.2. Sampling and biometrical analyses

At the end of the growth season of 2019, we sampled all (72 per treatment) sessile oak saplings to compare their biometric characteristics. Before harvesting, plant height (H, cm) and stem diameter at a height of 5 cm above the ground (D_0.05m_, mm) were recorded, and the cross-sectional area (CSA, cm^2^) was calculated using D_0.05m_. Then, ten fresh leaves were randomly selected from the upper, middle, and bottom parts of the crown of each harvested oak sapling, scanned, and oven-dried at 70 °C to measure their dry biomass using a precision scale (Radwag PS 6000.R1; precision: 0.01 g). Then, the specific leaf area (SLA; cm^2^ g^-1^) was calculated according to procedure described by Pietras et al. [42].

Subsequently, the above- and belowground biomass fractions of all samples were subdivided into leaves, branches, stems, and roots, which were further subdivided into coarse (> 2 mm) and fine roots (≤ 2 mm), based on root diameter. After measuring their fresh weight, the fractions were oven-dried at 70 (leaves) and 105 °C (woody biomass) to constant weight [57], and their dry biomass (g plant^-1^) were measured. Then, SLA and leaf biomass were used to calculate the leaf area (LA, m^2^ plant^-1^) of each plant.

### 2.3. Wood density

To determine the density of oven-dried wood, 5–8 cm long wood segments from a height of 10 cm above the ground were collected from all saplings and oven-dried (as described above). Their dry biomass and volume were measured using a digital scale (see above) and the water displacement method based on the Archimedes’ principle; briefly, a container filled with water was placed on the digital scale calibrated to the nearest 0.01 g and re-zeroed. The pith of each oven-dried wood segment was connected to a needle, and the segment was immersed into the water, ensuring that it did not touch the sides or bottom of the container. The weight of the water displaced was equal to the volume of the oven-dried segment, as 1 g of displaced water was equivalent to 1 cm^3^. Subsequently, the wood density (WD, g cm^-3^) of the oven-dried sample was calculated as the dry biomass of the wood sample divided by its volume [29].

### 2.4. Anatomical measurements

Immediately after harvesting, 1.8 mm thick microcores were collected from the base of the stems of 18 saplings per treatment using a Trephor increment borer [58]. Following the methodology of Fajstavr [59], the samples were dehydrated in successive baths of ethanol and embedded in paraffin. Then, 8–12 μm thick transverse wood sections were cut using a rotary microtome, stained in safranin and astra blue solutions, and mounted in Euparal. The cross-sections were then photographed using a digital video camera (Zeiss Axiocam 305 colour) attached to a light microscope (Zeiss Axio scope A1) at 100 × magnification.

Two to three sectors of the outermost ring, formed in 2019 (third year of CO_2_-enrichment experiment), from each cross-section were used to measure the width of the growth ring (RW) and the vessel lumen area (VLA) using ImageJ version 1.52a [60]. Cells with a lumen area ≤ 200 μm^2^ were excluded to distinguish xylem vessels from other cell types [61]. Then, average VLA, vessel density (VD; n mm^-2^), total vessel lumen area (TVA; μm^2^), and the proportion of TVA per analysed sector (PTVA; %) were calculated as described by Lotfiomran et al. [34]. The lumen diameter of each circular vessel was derived from VLA and used to calculate their hydraulically weighed diameter (D_hp_, μm) according to the equation: $D_{hp}=({\frac{\sum D^{4}}{N})}^{\frac{1}{4}}$, where D is the diameter of the vessel and N is the number of vessels [37]. The potential specific hydraulic conductivity of vessels (K_S_; kg m^-1^ s^-1^ MPa^-1^) was calculated using the Hagen–Poiseuille equation [62]: $K_{S}=\left(\frac{\pi \rho }{128\eta }\right)\;D_{hp}^{\;4}\;VD,$ where η is the viscosity of water (1,002 × 10^−9^ MPa s), ρ is the density of water (998.21 kg m^-3^), D_hp_ is the hydraulically weighed diameter of vessels, and VD is the mean vessel density. The potential hydraulic conductivity of a growth ring (K_ring_; kg m s^-1^ MPa^-1^) was estimated using the following equation by Noyer et al. [63]: K_ring_ = K_S_ × BAI_2019_, where the basal area increment of 2019 (BAI_2019_; cm^2^) was calculated using the equation BAI_2019_ = π (R^2^_2019_ − R^2^_2018_), where R is the radius of the tree in two subsequent years, 2018 and 2019 [64]. Subsequently, the xylem vulnerability index [65] was calculated by dividing the vessel diameter (D) by VD to obtain a rough estimation of the xeromorphic or mesomorphic character of the stem xylem.

According to the Hagen–Poiseuille law, large earlywood vessels in ring-porous oak species play a dominant role in axial water flow, wherein a few large earlywood vessels can transport an equal amount of sap as that transported by many small latewood vessels [37, 62]. However, the rationale of using all the vessels in the study was that the sampled wood sections had a semi-ring-porous to diffuse-porous structure instead of the ring-porous wood structure. In addition, the role of smaller vessels in ring-porous species is reportedly underestimated [66], considering their importance in tree survival when large vessels lose their transport capacity owing to drought-induced [67] or freeze-/thaw-induced embolism [68].

### 2.5. Statistical analyses

To assess the impact of CO_2_ concentration and fertilisation on sessile oak saplings, a split-plot experimental design was applied. After examining the normality assumption and variance homogeneity of the data, two-way analysis of variance (ANOVA) was used to evaluate the effects of CO_2_, nutrient supply, and their interactions on each measured biometrical and wood anatomical parameter. Additionally, Duncan’s method was used for the post-hoc comparisons of the treatment pairs. Non-normal data were transformed using a square root transformation and normality was then confirmed using the Kolmogorov–Smirnov test before performing the ANOVA.

Statistical analyses were performed using Statistica 13.0 (TIBCO Software Inc., Palo Alto, CA, USA), and statistical significance for all analyses was set at *p* ≤ 0.05. Boxplots were created using the software SigmaPlot^®^ 11.0 (Systat Software Inc., San Jose, CA, USA). Generalised additive models (GAMs) were fitted to each individual tree image using the mgcv package of R software [69], and average values were then calculated per treatment to evaluate the vessel-area size changes within the tree rings.

## 3. Results

### 3.1. Effects of eCO_2_ and nutrient supply on biometrical characteristics

**Fig 2:**
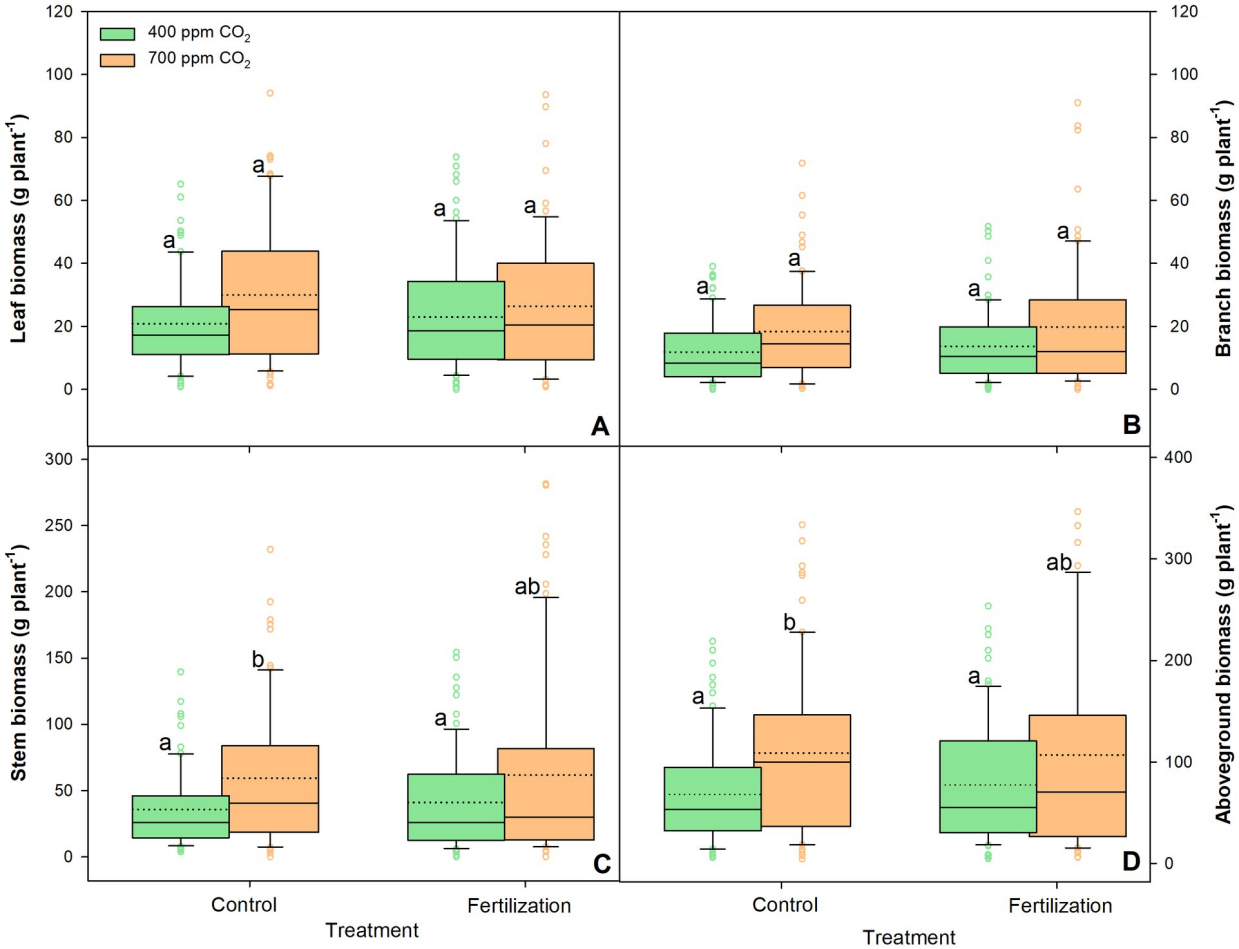
Changes in leaf biomass (A), branch biomass (B), stem biomass (C), and above-ground biomass (D) of sessile oak saplings (n = 72) treated under ambient (400 ppm CO_2_) and elevated (700 ppm CO_2_) and different nutrient supplies. The data are expressed as medians (solid lines) and means (dotted lines) of measurements. The box boundaries mark the 25^th^ and 75^th^ percentiles and whiskers the 10^th^ and 90^th^ percentiles. Circles marks outliners. Different letters indicate significant differences (p ≤ 0.05) estimated on the basis of Duncan’s ANOVA post-hoc test.

All aboveground morphological parameters measured were significantly affected by eCO_2_ treatment, whereas nutrient supply did not significantly affect them, except for SLA (*p* = 0.021) (Table 1). Furthermore, there was no significant effect of CO_2_ concentration–nutrient supply interactions on the aboveground biomass (Table 1). We observed a significant increase in all the biometrical parameters (H, D_0.05m_, and CSA_0.05m_) and all the aboveground biomass (i.e. leaves, branches, and stems) fractions (Fig 2, Table 1) under eCO_2_ treatment. Moreover, LA of the saplings was increased by 27%, and SLA was decreased by approximately 5.4% under eCO_2_ than under aCO_2_ (Table 1).

**Table 1:** Two-way analysis of variance (ANOVA) of the influence of CO_2_, nutrient supply and their interactions on sessile oak saplings aboveground and belowground biometrical characteristics.

	CO_2_		Nutrition		CO_2_ × Nutrition	
	F	p	F	P	F	p
Height (cm)	4.15	**0.043** ↑	0.438	0.509	0.001	0.975
D_0.05m_ (mm)	10.99	**<0.001** ↑	0.28	0.596	1.54	0.215
CSA_0.05m_ (mm)	13.92	**<0.001** ↑	0.16	0.686	1.22	0.270
Leaf biomass (g)	6.22	**0.014** ↑	0.16	0.691	0.03	0.862
Branch biomass (g)	4.67	**0.033** ↑	0.03	0.857	0.02	0.893
Stem biomass (g)	7.77	**0.006** ↑	0.77	0.381	0.18	0.673
Total aboveground biomass (g)	7.36	**0.007** ↑	0.45	0.501	0.06	0.809
Fine root (≤2 mm) biomass (g)	25.03	**<0.001** ↑	0.13	0.716	0.02	0.884
Coarse root (>2mm) biomass (g)	12.91	**<0.001** ↑	1.67	0.198	0.77	0.382
Total belowground biomass (g)	14.65	**<0.001** ↑	1.49	0.223	3.19	0.435
Total plant biomass (g)	11.58	**<0.001** ↑	0.07	0.985	0.53	0.467
WD (g/cm^3^)	8.70	**0.003** ↓	2.70	0.102	0.00	0.961
LA (m^2^/plant)	6.65	**0.011** ↑	0.02	0.889	0.85	0.356
SLA (cm^2^ g^-1^)	14.37	**<0.001** ↓	5.43	**0.021** ↓	0.75	0.390

D_0.05m_—diameter at 5 cm above ground; CSA_0.05m_—cross-sectional area at 5 cm above ground; WD—wood density; LA—leaf area; SLA—Specific leaf area;

^↑^—increase with eCO_2_;

^↓^—decrease with eCO_2_.

Statistically significant effects and interactions are indicated in bold (p ≤ 0.05).

**Fig 3:**
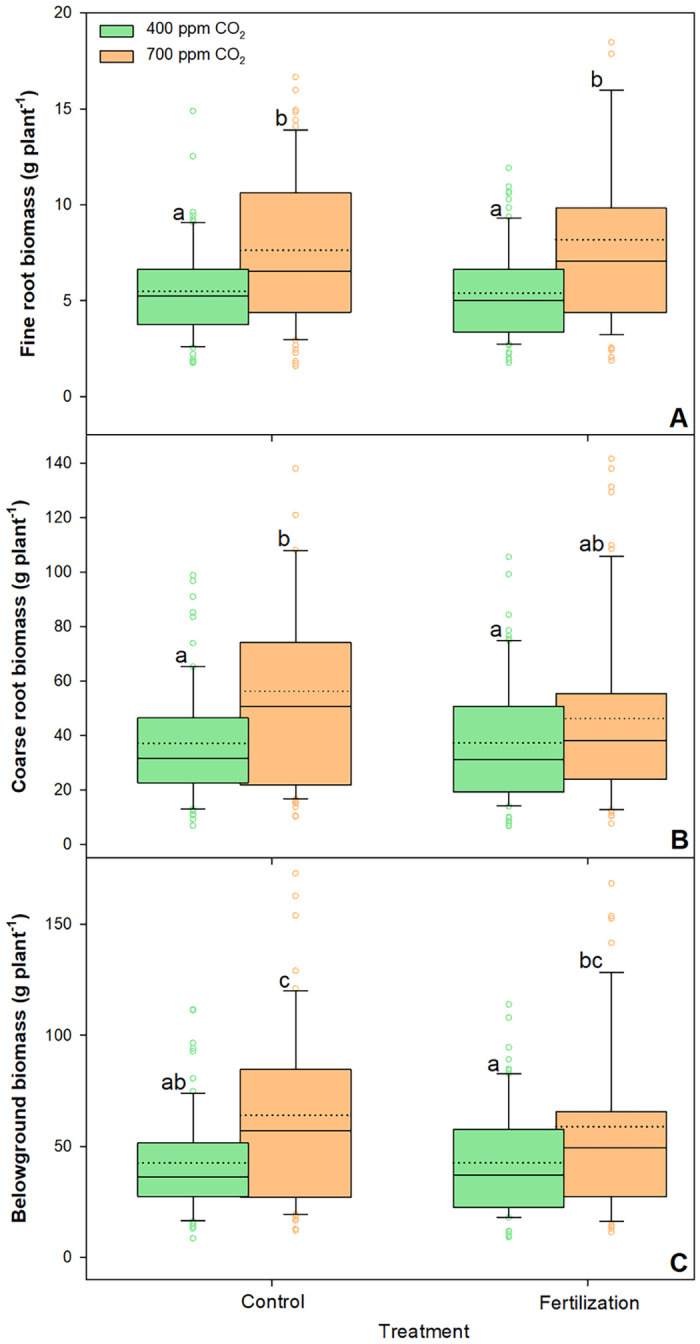
Changes in fine roots biomass (A), coarse roots biomass (B), and belowground biomass (C) of sessile oak saplings (n = 72) treated under ambient (400 ppm CO2) and elevated (700 ppm CO2) and different nutrient supplies. The data are expressed as medians (solid lines) and means (dotted lines) of measurements. The box boundaries mark the 25th and 75th percentiles and whiskers the 10th and 90th percentiles. Circles mark outliners. Different letters indicate significant differences (p ≤ 0.05) estimated on the basis of Duncan’s ANOVA post-hoc test.

Additionally, eCO_2_ had a positive effect on total sapling biomass, with an increase of 49% (Table 1). In turn, nutrient supply had a non-significant positive effect (an increase of 12%) on total sapling biomass under aCO_2_ treatment, whereas a non-significant negative effect (a decrease of 3%) was observed under eCO_2_ treatment (Figs 2 and 3, S1 Table).

Furthermore, eCO_2_ treatment had a positive effect on total belowground biomass, i.e. an increase of 47% in fine and coarse roots (Table 1). However, nutrient supply had a negative effect on both fine and coarse roots (Fig 3). The saplings growing under eCO_2_ treatment had significantly higher amounts of fine roots (45%) than those growing under aCO_2_ treatment.

### 3.2. Effects of eCO_2_ and nutrient supply on wood structure

**Fig 4:**
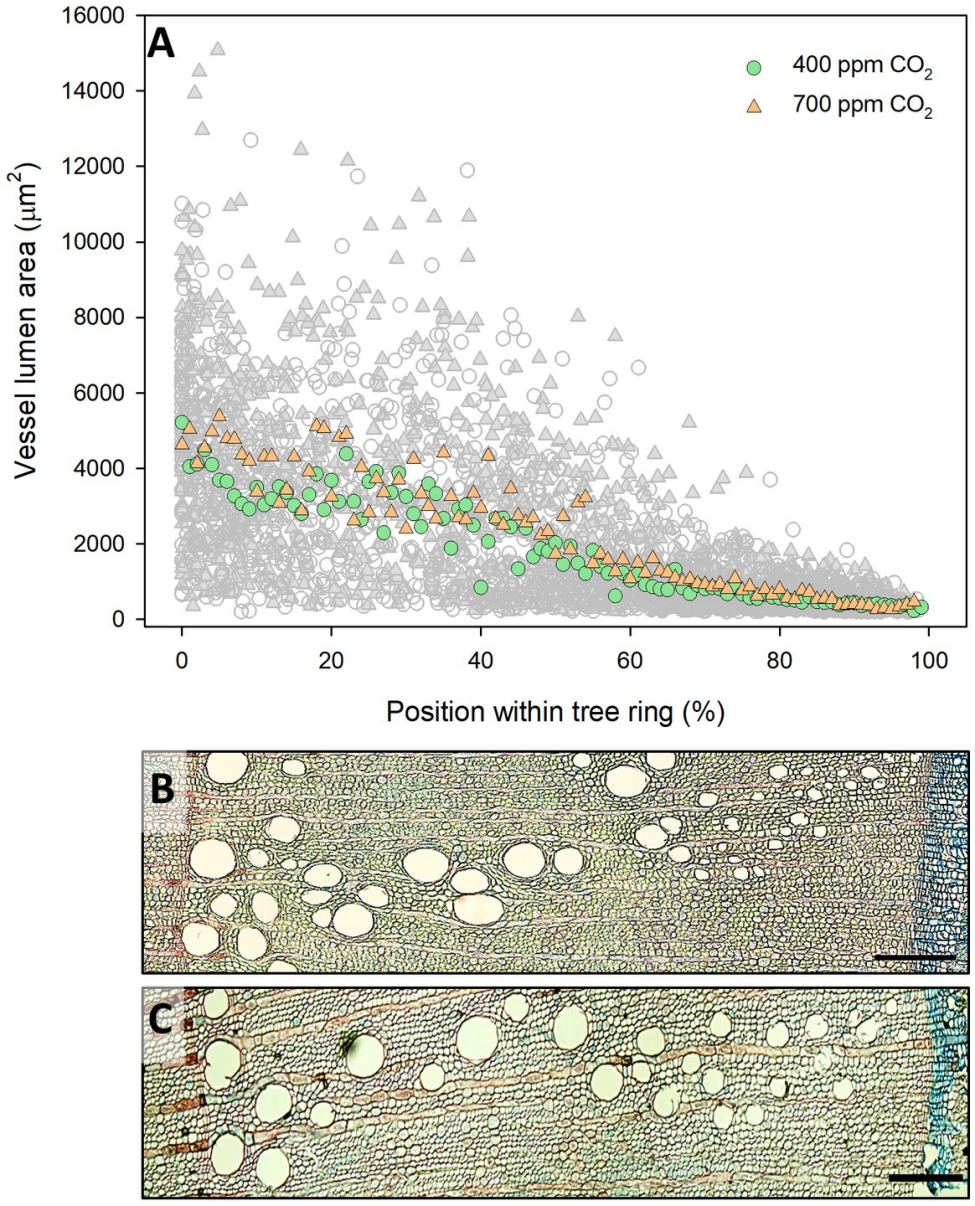
Overview of tree ring in 2019. (A) The relative position of vessel lumen areas within tree ring created in 2019 with applied GAMs denoted by orange (elevated CO_2_) and green characters (ambient CO_2_). Microscope images of a cross-section of the 2019 annual ring of a sessile oak saplings growing in (B) ambient CO_2_ glass dome and (C) elevated CO_2_ glass dome. Scale bars = 200 μm.

All wood anatomical parameters showed significant differences under eCO_2_ treatment, except for TVA, PTVA, and K_s_, whereas neither nutrient supply nor the interaction between CO_2_ concentration and nutrient supply showed any influence on wood structure, except for BAI (Table 2). Larger vessels were observed in the earlywood and transition zones under eCO_2_ treatment (tree-ring position <50%), while latewood zone vessels tended to display similar lumen area regardless of the CO_2_ treatments (Fig 4).

**Table 2:** Two-way analysis of variance (ANOVA) of the influence of CO_2_, nutrient supply, and their interactions on sessile oak saplings anatomical characteristics.

	CO_2_		Nutrition		CO_2_ × Nutrition	
	F	p	F	p	F	p
Vessel diameter (μm)	8.06	**0.006** ↑	0.34	0.560	0.42	0.519
VLA (μm^2^)	9.32	**0.003** ↑	0.69	0.410	0.74	0.394
TVA (μm^2^)	0.56	0.455	0.34	0.560	1.49	0.226
PTVA (%)	0.56	0.459	0.58	0.450	0.25	0.621
D_hp_ (μm)	8.69	**0.004** ↑	0.49	0.484	2.19	0.143
RW (μm)	8.31	**0.005** ↑	0.50	0.483	0.02	0.891
BAI (mm^2^)	11.61	**0.001** ↑	0.53	0.471	4.20	**0.044**
VD (N_o_ mm^-2^)	5.43	**0.023** ↓	0.59	0.443	0.62	0.434
K_s_ (kg m^-1^ s^-1^ MPa^-1^)	0.46	0.499	0.13	0.722	0.40	0.531
K_ring_ (kg m s^-1^ MPa^-1^)	7.88	**0.007** ↑	0.55	0.461	2.44	0.123
Vulnerability index	14.57	**0.001** ↑	1.73	0.192	1.27	0.263

VLA—Vessel lumen area; TVA—total lumen vessel area; PTVA—the proportion of the total vessel lumen area per analyzed sector; D_hp_ -hydraulic weighted diameter; RW—ring width; BAI—Basal area increment; VD—Vesel density; K_s_—potential specific hydraulic conductivity; K_ring_—potential hydraulic conductivity for a growth ring; Vulnerability index was calculated after Carlquist [65].

^↑^—increase with eCO_2_;

^↓^—decrease with eCO_2_.

Statistically significant effects and interactions are indicated in bold (p ≤ 0.05).

**Fig 5:**
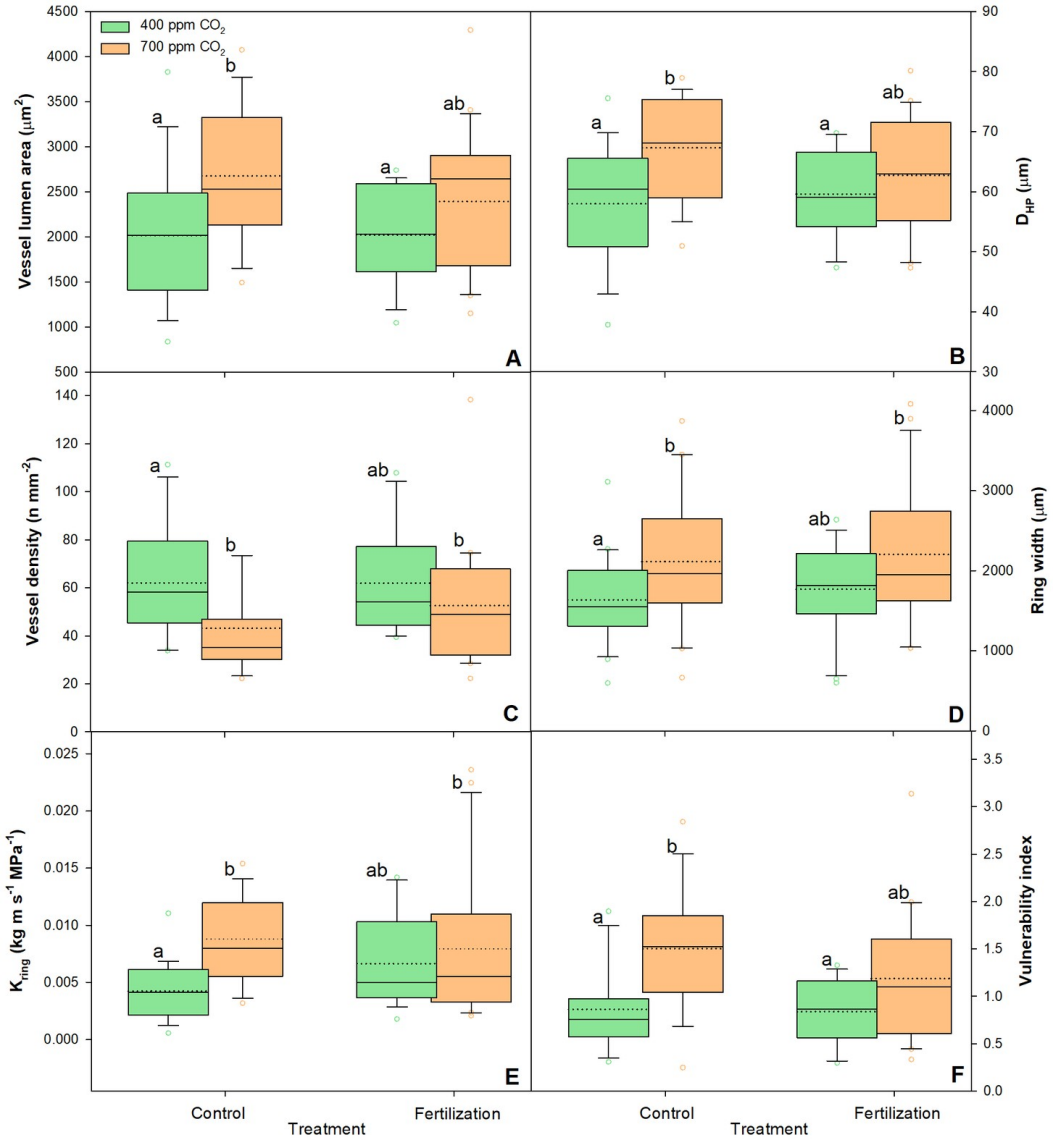
Changes in vessel lumen area (A), D_HP_ (B), vessel density (C), Ring width (D), K_ring_ (E), and Vulnerability index (F) of sessile oak saplings (n = 18) treated under ambient (400 ppm CO_2_) and elevated (700 ppm CO_2_) and different nutrient supplies. The data are expressed as medians (solid lines) and means (dotted lines) of measurements. The box boundaries mark the 25^th^ and 75^th^ percentiles and whiskers the 10^th^ and 90^th^ percentiles. Circles mark outliners. Different letters indicate significant differences (p ≤ 0.05) estimated on the base of Duncan’s ANOVA post-hoc test.

The VLA was significantly increased at eCO_2_, whereas VD was significantly lower (Fig 5, Table 2), with the highest VLA and lowest VD recorded in the saplings under eCO_2_ treatment without nutrient supply (Fig 5, S2 Table). In contrast, TVA and PTVA showed no significant differences under eCO_2_ as compared with aCO_2_ (Table 2, S2 Table), and RW exhibited the largest increase under the nutrient supply plus eCO_2_ combination treatment (Fig 5, S2 Table).

The values of all the traits associated with hydraulic conductivity increased under eCO_2_ (Table 2), and the highest statistically significant values of D_hp_ were observed in saplings under eCO_2_ without nutrient supply (Fig 5, S2 Table). The K_s_ values were not significantly different between any of the treatments (S2 Table), whereas K_ring_ values were significantly higher in saplings grown at eCO_2_ than in the saplings grown at aCO_2_, regardless of nutrient supply (Fig 5, S2 Table). Furthermore, the vulnerability index was significantly higher in the former case (Table 2), with the highest value observed in unfertilised saplings (Fig 5, S2 Table).

**Fig 6:**
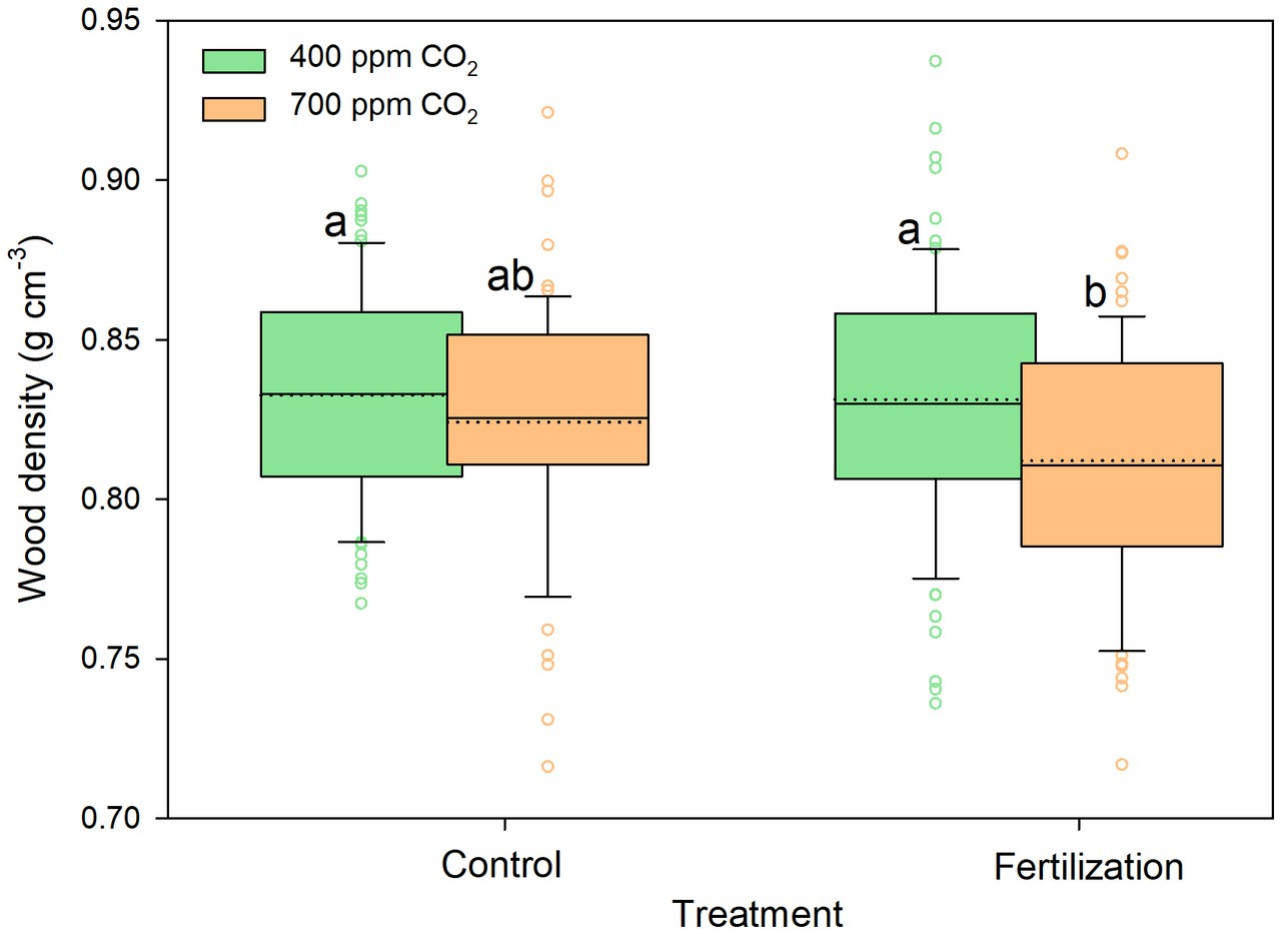
Changes in over-dry wood density of sessile oak saplings (n = 72) treated under ambient (400 ppm CO_2_) and elevated (700 ppm CO_2_) and different nutrient supplies. The data are expressed as medians (solid lines) and means (dotted lines) of measurements. The box boundaries mark the 25^th^ and 75^th^ percentiles and whiskers the 10^th^ and 90^th^ percentiles. Circles mark outliners. Different letters indicate significant differences (p ≤ 0.05) estimated on the base of Duncan’s ANOVA post-hoc test.

Overall, the saplings grown under eCO_2_ concentration treatment had significantly lower densities (approximately 1.7%); however, nutrient supply or the CO_2_-concentration—nutrient -supply interaction had no effect (Table 1). Saplings with nutrient supplementation had lower wood densities than the saplings grown without nutrient supply under the two different CO_2_ concentrations tested here; furthermore, the saplings grown with nutrient supply under eCO_2_ exhibited significantly lower wood densities than those in any other treatment (Fig 6, S1 Table).

## 4. Discussion

### 4.1. Effects of eCO_2_ on biometrical characteristics of sessile oak saplings

Our study revealed a high growth benefit for sessile oak saplings grown in the eCO_2_ environment. After three years of exposure to eCO_2_ concentration treatment, the saplings grew taller and wider stems and accumulated 49% more dry biomass (both above- and belowground) than the saplings grown under aCO_2_ concentration treatment (Figs 2 and 3, Table 1). Previous studies have shown growth stimulation in young saplings of many different tree species in response to eCO_2_ [10, 70]. In sessile oak saplings grown under eCO_2_, Ofori-Amanfo et al. [15] revealed an increase in the light-saturated CO_2_ assimilation rate, a decrease in stomatal conductance, and consequently improved WUE. Evidently, eCO_2_ concentrations directly influence plant growth by increasing photosynthesis and decreasing stomatal conductance, resulting in a subsequent increase in WUE and carbohydrate availability for growth, eventually leading to greater biomass production [12]. Consistent with our results, Saxe et al. [71] reported that eCO_2_ significantly increased plant size and aboveground biomass, with angiosperms responding less (average increase of 49%) than conifers, which showed an average biomass increase of 130% (Figs 2 and 3, Table 1 and S1 Table).

Increased plant productivity is directly related to greater LA, which is crucial for radiation interception, photosynthetic assimilation, and, ultimately, plant growth and performance. Previous studies have reported larger LA and leaf biomass and reduced SLA in plants growing under eCO_2_ [11]. This study also showed that LA increased by 27% and SLA decreased by 5.4% in saplings grown under eCO_2_ treatment (Table 1 and S1 Table). Evidence suggest that eCO_2_ affects plant cell proliferation by enhancing cell division and cell enlargement [30], although, according to Pritchard et al. [11], increased leaf growth under eCO_2_ conditions is the result of cell proliferation stimulated by cell enlargement rather than cell division. Cell enlargement under eCO_2_ levels is achieved by the decreasing osmotic potential in the cell driven by excess carbohydrates in the sap, which in turn promotes further water intake by the cell, thereby increasing turgor-driven cell wall enlargement [72]. Nevertheless, under eCO_2_ conditions, the properties of the cell wall might be altered, and the cells may stretch to a greater extent than cells developing under lower CO_2_ concentrations [73].

However, the tree response to eCO_2_ is rather complex and we need to take into account complex interactions among CO_2_ concentrations, photosynthesis and other environmental factors, as well as even more complex relationships among carbon assimilation, plant respiration and growth [9, 10, 16]. For example, mature trees do not show greater biomass production under eCO_2_ despite the stimulated photosynthesis [19] suggesting that C availability itself is not necessarily the most limiting factor for tree growth [74, 75]. Instead, it was suggested that growth is limited by environment, and in particular water stress limits the ability of cambium to convert the carbon into growth [76]. In fact, many studies on different plant species have shown that during heat and water stress, C demand for growth always declines before photosynthesis (carbon supply) is affected [74, 75]. However the surplus in carbon is usually allocated in different sink other than growth (biomass), such as respiration [9, 19]. Thus, climate changes are widely expected to increase more extreme weather events, in particular heat and drought stress [44, 45], which will certainly mitigate the potential of eCO_2_ to stimulate tree growth in many regions [9].

### 4.2. Effects of eCO_2_ on wood structure of sessile oak saplings

Yazaki et al. [31] reported that both enhanced cell division and cell enlargement were responsible for increased wood formation under eCO_2_ conditions. In this study, sessile oak saplings grown under eCO_2_ treatment exhibited significantly increased RW, BAI, VLA, and, consequently, xylem hydraulic conductance (Figs 4 and 5, Table 2 and S2 Table). Wood formation is affected by cambial activity, which in turn may be affected by eCO_2_ concentrations. Consistently, Watanabe et al. [36] reported a high number of active cambium cells in two ring-porous species under eCO_2_ conditions, implying that the rate of cell division might be affected under varying CO_2_ concentrations [5]. Moreover, the systematic review by Yazaki et al. [26] reported that the diameter and number of angiosperm xylem conduits increased owing to the changes in the inner water balance induced by eCO_2_. This was confirmed in the ring-porous *Q*. *robur* saplings, where both size and density of the vessels increased under eCO_2_, resulting in a two-fold increase in TVA [35]. The results of our study are partly in agreement with the findings of the above study, because we observed an increased VLA and a significantly decreased VD in the saplings grown under eCO_2_, whereas TVA remained unchanged (Fig 5, Table 2).

Although information on the effects of eCO_2_ conditions on WD is limited and the results of previous studies are inconsistent [5, 26, 29, 30], eCO_2_ conditions reputedly affect the duration or extent of secondary cell wall deposition in addition to cell division and enlargement [5, 26, 77]. Domec et al. [30] reported that photosynthesis stimulation under eCO_2_ yielded sufficient building material for both thicker cell walls and sustained cell wall enlargement, which simultaneously increased WD and xylem conductivity. However, these findings are partly in agreement with those of Yazaki et al. [31], who suggested a major effect of eCO_2_ concentrations on cell division and enlargement rather than on cell wall deposition, resulting in a low WD in *L*. *sibirica* saplings growing under eCO_2_ treatment. The findings of Yazaki et al. [31] are consistent with our study results, which revealed a significantly low WD in saplings growing under eCO_2_ (Fig 6, Table 1).

The tree ring structure, defined by the number and dimensions of the constituent cells, is the primary determinant of WD. Angiosperm wood is a complex tissue that comprises vessels to conduct water, fibres to provide mechanical support, and parenchyma cells to store nutrients; furthermore, their relative proportions in the wood influences WD [39]. Vessels of ring-porous tree species have wider lumens than those in other wood cells. Moreover, vessel lumens have zero density and consequently negatively affect WD [78, 79]. However, we could not verify this observation in this study, as no statistical differences in TVA or PTVA were observed between saplings grown under the aCO_2_ and eCO_2_ treatments (Table 2 and S2 Table). Therefore, other anatomical characteristics, such as fibres, living parenchyma [39], and cell wall thickness of each cell type [77] may be responsible for the low WD in the saplings grown in an eCO_2_ environment. As this study was limited to wood anatomy of the conductive tissue in the last formed ring, we cannot draw any definite conclusions regarding this issue.

Moreover, studies by Bergès et al. [53] and Pretzsch et al. [41] reported long-term increased growth and reduced WD in sessile oak trees using dendrochronological and long-term inventory data, respectively, which are in agreement with our observations. Additionally, Pretzsch et al. [41] suggested that N supply via atmospheric deposition might be a major reason for a decrease in WD, rather than the increase in CO_2_ concentration, which contradicts our results (Table 1). Nevertheless, although we observed the highest WD decrease in the saplings grown with nutrient supplementation under the eCO_2_ treatment (Fig 6), we unexpectedly did not find significant effects of the interaction between CO_2_ concentration and nutrient supply (Table 1).

### 4.3. Response of sessile oak saplings to nutrient supply

Neither nutrient supply nor its interaction with CO_2_ concentration exhibited any significant effect on the experimental saplings, except for SLA (Tables 1 and 2). Similar results were reported by Lotfiomran et al. [34], who found that the anatomical features in *F*. *sylvatica* saplings were more strongly affected by eCO_2_ than by fertilisation. Similarly, although eCO_2_ enhanced the growth of *Quercus alba* saplings on N-limited soils, tissue N concentrations were significantly reduced compared to those in saplings grown under aCO_2_ treatment [80]. This implies that plants under eCO_2_ concentration were highly efficient, with less investment in their tissue [81], which was indirectly verified in our study via the reduced WD measured in the saplings grown under eCO_2_ treatment (Table 1).

The increase in sapling growth observed in our study required sufficient nutrients to take advantage of the eCO_2_ condition; that increased nutrient demand was met by the relatively fertile native soil where the saplings were planted. It has been demonstrated that eCO_2_ conditions accelerate the effects of nutrient unavailability with time [21–23]. For instance, Rolo et al. [82] reported that the enhanced growth in *F*. *sylvatica* and *Picea abies* Karst. saplings induced by eCO_2_ rendered the soil nutrient-deficient after six years. Therefore, we hypothesise that nutrient limitation in the glass dome supplied with eCO_2_ treatment will develop gradually and eventually hinder the CO_2_-induced increase in plant biomass productivity [21].

### 4.4. Possible implications on future forest management

Higher biomass production in sessile oak saplings under eCO_2_ treatment was supported by an efficient xylem hydraulic system (Tables 1 and 2). However, the trade-off between hydraulic efficiency and safety (the ability of xylem to resist the formation and spread of embolisms) suggest that xylem comprising larger vessels may be less resistant to embolism during severe drought [83]. Similar to our findings, Levanic et al. [84] observed higher D_hp_, BAI, and consequently K_ring_ in dead *Q*. *robur* trees than in the trees that survived a drought period, suggesting that the trees which died had been hydraulically maladjusted for dry conditions. This was also verified by the higher vulnerability index [65] observed in the saplings grown under eCO_2_ in our study (Fig 5, Table 2). According to the principles of Carlquist’s vulnerability index, saplings with wider and less abundant vessels express higher vulnerability indices and a higher mesic character, which are reflected in xylem conduits that are less resistant to embolism. However, enhanced WUE and larger absorptive area of the root system under eCO_2_, indicated by the increased biomass of both fine and coarse roots (Fig 3, Table 1), might compensate for xylem susceptibility and increase drought-resistance in sessile oak saplings [15]. Nevertheless, root capacity for water-uptake depends not only on root mass but also rooting depth as well as the area and activity of fine roots [11].

In this study, we found a significant effect of eCO_2_ treatment on WD (Fig 6, Table 1), which is reportedly associated with many wood characteristics [39]. Low WD is well associated with reduced wood stiffness and strength [85], as has been observed in conifer and angiosperm saplings growing under eCO_2_ conditions [29, 77], and higher susceptibility to wind and snow [43]. However, tree performances in response to mechanical constraints, such as wind or snow, are driven mainly by tree allometry, where the increase in stem diameter improves the fourth power resistance of stem to bending [86, 87] and proportionally to another mechanical stability trait [88]. Our results suggested that the mechanical performances are relayed by the improved growth rate under eCO_2_ and that the decrease in WD reflects the adjustments of the hydraulic system and management of construction cost to the increasing stem volume. Nevertheless, storm-mediated forest damage has increased in recent decades [89]. As suggested by Pretzsch et al. [41], although changes in forest management are primarily responsible for these observations, low mechanical stability—as indicated by lower WD values—might also contribute to forest damage.

Our study focused on juvenile wood, and it is uncertain how mature trees will respond to future atmospheric conditions, though correlations between the qualities of juvenile and mature wood have been reported [90]. However, Pretzsch et al. [41] reported that unlike older trees with denser and more stable wood, young saplings growing under conditions leading to lower WD and less mechanical stability will be more affected by high winds [43]. Moreover, a recent study on tree ring-growth in many species and environments found that faster tree ring-growth directly reduces tree lifespan [91].

Therefore, despite the increased biomass production at eCO_2_ in this economically and ecologically important European tree species, the impacts of lower density on mechanical strength, and xylem becoming more vulnerable to drought, may lead to earlier mortality offsetting the positive effect of future eCO_2_.

## Supporting information

S1 Table Biometric characteristics of (*Quercus petraea* (Matt.) Liebl.) saplings under different CO_2_ concentrations and nutrient supplies. The data represent mean (± standard error of the mean). Different letters indicate significant differences (p≤0.05) estimated on the basis of Duncan’s ANOVA post-hoc test. D_0.05m_—diameter at 5 cm above ground; CSA_0.05m_—cross-sectional area at 5 cm above ground; LA—leaf area; SLA—Specific leaf area.
(DOCX)

S2 Table Vessel anatomy characteristics of (*Quercus petraea* (Matt.) Liebl.) saplings under different CO_2_ concentrations and nutrient supplies. The data represent mean (± standard error of the mean). Different letters indicate significant differences (p≤0.05) estimated on the basis of Duncan’s ANOVA post-hoc test. TVA—Total vessel lumen area; PTVA—the proportion of the total vessel lumen area per analyzed sector; D_hp_—hydraulic diameter; VD—vessel density; TRW_2019_—Tree ring width; BAI_2019_—Basal area increment; K_s_—Potential specific hydraulic conductivity; K_ring_—potential hydraulic conductivity for a growth ring; VI—Vulnerability index calculated after Carlquist [65].
(DOCX)
