# Supplementary material for: Increased wood biomass growth is associated with lower wood density in Quercus petraea (Matt.) Liebl. saplings growing under elevated CO2
Source: PLoS One. 2021 Oct 22;16(10):e0259054. doi: 10.1371/journal.pone.0259054 (PMC8535391; doi:10.1371/journal.pone.0259054)
Supplement: S1 Table — The data represent mean (± standard error of the mean). Different letters indicate significant differences (p≤0.05) estimated on the basis of Duncan’s ANOVA post-hoc test. D0.05m—diameter at 5 cm above ground; CSA0.05m—cross-sectional area at 5 cm above ground; LA—leaf area; SLA—Specific leaf area. (DOCX) [file pone.0259054.s001.docx]

**S1 Table**. Biometric characteristics of (Quercus petraea (Matt.) Liebl.) saplings under different CO_2_ concentrations and nutrient supplies

|  | 400 ppm _a_CO_2_ | | 700 ppm _e_CO_2_ | |
| --- | --- | --- | --- | --- |
|  | Control | Nutrition | Control | Nutrition |
| Height (cm) | 108.13 (±5.05)^a^ | 114.89 (±6.25)^ab^ | 128.91 (±6.55)^a^ | 126.32 (±7.88)^ab^ |
| D_0.05m_ (mm) | 11.29 (±0.41)^a^ | 11.65 (±0.44)^a^ | 13.59 (±0.55)^b^ | 12.69 (±0.61)^b^ |
| CSA_0.05m_ (mm^2^) | 109.15 (±7.80)^a^ | 116.66 (±8.70)^ab^ | 160.98 (±12.06)^c^ | 144.80 (±13.58)^bc^ |
| Leaf biomass (g) | 20.19 (±1.75)^a^ | 22.59 (±2.16)^a^ | 29.33 (±2.50)^a^ | 27.67 (±2.66)^a^ |
| Branch biomass (g) | 12.15 (±1.21)^a^ | 13.62 (±1.35)^a^ | 18.51 (±1.90)^a^ | 17.93 (±1.89)^a^ |
| Stem biomass (g) | 35.46 (±3.54)^a^ | 40.39 (±4.29)^a^ | 60.92 (±6.35)^b^ | 53.19 (±7.07)^ab^ |
| Above-ground biomass (g) | 67.79 (±6.23)^a^ | 76.61 (±7.45)^a^ | 108.77 (±10.11)^b^ | 98.80 (±11.20)^ab^ |
| Fine roots (≤2 mm) biomass (g) | 5.49 (±0.29)^a^ | 5.32 (±0.30)^a^ | 7.86 (±0.52)^b^ | 7.89 (±0.64)^b^ |
| Coarse roots (>2mm) biomass (g) | 37.05 (±2.48)^a^ | 36.74 (±2.78)^a^ | 56.61 (±4.87)^b^ | 48.84 (±4.93)^ab^ |
| Below-ground biomass (g) | 42.53 (±2.70)^ab^ | 42.06 (±2.99)^a^ | 64.48 (±5.26)^c^ | 56.73 (±5.49)^bc^ |
| Total plant biomass (g) | 110.76 (±8.35)^a^ | 124.40 (±11.24)^ab^ | 177.96 (±16.10)^c^ | 171.77 (±19.72)^bc^ |
| Oven-dry wood density (g/cm^3^) | 0.86 (±0.004)^a^ | 0.85 (±0.004)^a^ | 0.84 (±0.005)^ab^ | 0.84 (±0.005)^b^ |
| LA (m^2^/plant) | 0.30 (±0.03)^a^ | 0.33 (±0.03)^ab^ | 0.42 (±0.03)^b^ | 0.38 (±0.04)^ab^ |
| SLA (cm^2^ g^-1^) | 151.88 (±2.13)^a^ | 145.44 (±1.90)^b^ | 142.49 (±1.61)^ab^ | 139.55 (±2.34)^c^ |

The data represent mean (± standard error of the mean). Different letters indicate significant differences (p≤0.05) estimated on the basis of Duncan's ANOVA post-hoc test. D_0.05m_ - diameter at 5 cm above ground; CSA_0.05m_ - cross-sectional area at 5 cm above ground; LA - leaf area; SLA - Specific leaf area.
