# Supplementary material for: Increased wood biomass growth is associated with lower wood density in Quercus petraea (Matt.) Liebl. saplings growing under elevated CO2
Source: PLoS One. 2021 Oct 22;16(10):e0259054. doi: 10.1371/journal.pone.0259054 (PMC8535391; doi:10.1371/journal.pone.0259054)
Supplement: S2 Table — The data represent mean (± standard error of the mean). Different letters indicate significant differences (p≤0.05) estimated on the basis of Duncan’s ANOVA post-hoc test. TVA—Total vessel lumen area; PTVA—the proportion of the total vessel lumen area per analyzed sector; Dhp—hydraulic diameter; VD—vessel density; TRW2019—Tree ring width; BAI2019—Basal area increment; Ks—Potential specific hydraulic conductivity; Kring—potential hydraulic conductivity for a growth ring; VI—Vulnerability index calculated after Carlquist [65]. (DOCX) [file pone.0259054.s002.docx]

**S2 Table**. Vessel anatomy characteristics of (Quercus petraea (Matt.) Liebl.) saplings under different CO_2_ concentrations and nutrient supplies

|  | 400 ppm _a_CO_2_ | | 700 ppm _e_CO_2_ | |
| --- | --- | --- | --- | --- |
|  | Control | Nutrition | Control | Nutrition |
| Vessel diameter (µm) | 57.87 (±2.18)^a^ | 54.67 (±1.69)^a^ | 65.95 (±2.34)^b^ | 60.00 (±2.16)^a^ |
| Vessel lumen area (µm²) | 2973.67 (±204.5)^a^ | 2703.73 (±143.3)^a^ | 3829.65 (±247.1**)**^b^ | 3215.36 (±212.9)^ab^ |
| TVA (µm²) | 81923.20 (±8371.96)^a^ | 98349.68 (±8819.74)^a^ | 99859.67 (±8016.08)^a^ | 94073.80 (±10381.53)^a^ |
| PTVA (%) | 11.36 (±0.75)^a^ | 11.58 (±0.56)^a^ | 10.31 (±0.93)^a^ | 11.37 (±1.02)^a^ |
| D_hp_ (µm) | 65.52 (±2.30)^a^ | 64.61 (±1.63)^a^ | 74.35 (±2.38)^b^ | 68.67 (±2.21)^ab^ |
| VD (N_o_ mm^-2^) | 37.07 (±3.98)^a^ | 42.34(±4.00)^ab^ | 25.77(±2.86)^b^ | 32.85(±2.36)^b^ |
| RW (µm) | 1635.67(±117.19)^a^ | 1768.77(±128.51)^ab^ | 2112.97(±189.65)^b^ | 2202.69 (±185.89)^b^ |
| BAI_2019_ (mm^2^) | 242.99 (±39.33)^a^ | 351.53 (±39.33)^ab^ | 456.53(±40.47)^b^ | 404.69 (±37.31)^b^ |
| K_s_ (kg m^-1^ s^-1^ MPa^-1^) | 15.99 (±1.42)^a^ | 17.59 (±1.72)^a^ | 17.95 (±1.31)^a^ | 17.39 (±1.44)^a^ |
| K_ring_ (kg m s^-1^ MPa^-1^) | 0.0043 (±0.0011)^a^ | 0.0067 (±0.0011)^ab^ | 0.0088 (±0.0011)^b^ | 0.0079 (±0.0009)^b^ |
| VI | 0.86 (±0.11)^a^ | 0.84 (±0.08)^a^ | 1.50 (±0.15)^b^ | 1.19 (±0.15)^ab^ |

The data represent mean (± standard error of the mean). Different letters indicate significant differences (p≤0.05) estimated on the basis of Duncan's ANOVA post-hoc test.

TVA - Total vessel lumen area; PTVA – the proportion of the total vessel lumen area per analyzed sector; D_hp_ - hydraulic diameter; VD - vessel density; TRW_2019_ - Tree ring width; BAI_2019_ - Basal area increment; K_s_ - Potential specific hydraulic conductivity; K_ring_ - potential hydraulic conductivity for a growth ring; VI - Vulnerability index calculated after Carlquist [1].

References:

1. Carlquist S. Ecological Factors in Wood Evolution: A Floristic Approach. Am J Bot. 1977. doi:10.2307/2442382
